# Anxiety and depression among HIV patients of the infectious disease department of Conakry University Hospital in 2018

**DOI:** 10.1017/S095026881900222X

**Published:** 2020-01-14

**Authors:** A. Camara, M.S. Sow, A. Touré, F.B. Sako, I. Camara, K. Soumaoro, A. Delamou, M. Doukouré

**Affiliations:** 1 Department of Public Health, Faculty of Science and Technical of health, Gamal Abdel Nasser University, Conakry, Guinea; 2 Department of Infectious and Tropical Diseases, Conakry University Hospital, Guinea; 3 Department of psychiatry, Conakry University Hospital, Guinea

**Keywords:** Anxiety, depression, Guinea, HIV/AIDS

## Abstract

Anxiety and depression continue to be significant comorbidities for people with human immunodeficiency virus (HIV) infection. The aim of this study was to determine the prevalence of anxiety and depression disorder among HIV patients at Conakry, Guinea. In this cross-sectional study, we described socio-demographic, clinical and psychosocial data related to anxiety and depression in 160 HIV patients of the University Teaching Hospital, Conakry, Guinea. The Hospital Anxiety and Depression Scale (HADS) was used for measuring depression and anxiety in the prior month. The HADS score of ⩾8 was used to identify possible cases of depression and anxiety. Multivariate logistic regression analyses were performed to identify factors associated with symptoms of anxiety and depression. The prevalence of comorbid depression and anxiety among HIV patients was 8.1% and the prevalence of anxiety and depressive symptoms among HIV-infected patients was 13.8% and 16.9%, respectively. Multivariate analysis showed that individuals having BMI ⩽ 18 (AOR = 3.62, 95% confidence interval (CI) 1.37–9.57) and who did not receive antiretroviral treatment (AOR = 18.93, 95% CI 1.88–188.81) were significantly more likely to have depressive symptoms. Similarly, having age <40 years (AOR = 2.81, 95% CI 1.04–7.58) was also significantly associated with anxiety. Prevalence of symptoms of anxiety and depression was high in these HIV patients. This suggests a need for training on the screening and management of anxiety and depression among HIV patients.

## Introduction

Depression is responsible for more ‘years lost’ to disability than any other condition. According to the World Health Organization (WHO), 350 million suffer from depression [1]. One review found that patients tended to experience depression or anxiety as a consequence of being diagnosed with a chronic disease [2]. Depression is a common mental disorder that presents with depressed mood, decreased energy, disturbed sleep or appetite, loss of interest or pleasure, feelings of guilt or low self-worth, and poor concentration [3]. Anxiety is a vague, subjective, non-specific feeling of uneasiness, fears, tension, apprehension and a sense of impending doom, irrational avoidance of objects or situations and anxiety attack [4]. Anxiety was associated with gender, age and chronic diseases [5, 6].

Importantly, human immunodeficiency virus (HIV)/AIDS and anxiety/depression are interlinked. Over half of all HIV-infected individuals suffer from mental health disorders [7] and depression and anxiety disorders are more common in HIV-infected individuals than in the general population [8, 9]. Worldwide, 36.9 million people were living with HIV. In 2017, there were an estimated 1.8 million (1.4–2.4 million new HIV infections and 940 000 deaths due to AIDS [10]). National prevalence of HIV in Guinea was 1.6% in 2018 [11]. People suffering from major anxiety/depression may be more likely at risk to contract HIV, have reduced adherence, impair their immune function, increase health cost disability and mortality [12, 13]. Conversely, an HIV+ diagnosis may trigger symptoms of anxiety and depression [14, 15], which could once again lead to risky sexual behaviour and the spreading of the virus. In addition, studies have shown that people suffering from depression are less likely to adhere to treatment for both mental illness and for antiretroviral treatment (ART) [16, 17]. Unfortunately, more than half of the HIV+ population that suffer from depression have not received an official diagnosis of their depression [18]. There are currently no guidelines to manage psychiatric disorders in the HIV clinic setting in Guinea. In this study, the objectives were to determine the prevalence of symptoms of depression and anxiety and to identify risk factors in HIV-infected patients in Guinea.

## Methods

### Study setting and design

An institution-based cross-sectional study was conducted at the University Hospital, in the Department of Infectious and Tropical Diseases in Conakry, Guinea. The study was conducted between December 2017 and April 2018.

### Study population

The study population consisted of all adult HIV patients who were on follow-up at the infectious diseases department at University Hospital, including both ambulatory and hospitalised patients. The sample was composed of patients over 18 years old of both genders. Those HIV patients with difficulties answering the questionnaire properly, such as the ones with hearing, speech or mental impairment and non-consenting were excluded from the study.

### Sampling procedures

Sample size was determined based on the single population proportion formula using Epi-info version 7 with a 95% confidence interval (CI), 5% margin of error and assuming a prevalence of depression to be 9.7% (19). Assuming a 10% non-response rate, a total sample size of 160 HIV cases was required. Systematic sampling technique was used to select from amongst ambulatory patients. The sampling interval was determined by dividing the total study population (*n* = 600) of patients who had follow-up during the 3 months data collection period by the target sample size (*n* = 160), with a random starting point. During the last months preceding the study, 600 outpatients had been seen and thus the sampling interval was estimated to be 4.

In addition, all hospitalised patients during the study period were invited to participate in the study.

### Data and measures

Data were collected using pretested interviewer-administered questionnaires, which contained questions on socio-demographic characteristics (age, gender, education, occupation, marital status, education level, dwelling type and others), clinical characteristics (time since first HIV test, CD4 count, started ART, temporary stop of ART at any point in the last year) and symptoms of depression and anxiety. Symptoms of anxiety and depression were measured using the Hospital Anxiety and Depression Scale (HADS) [20]. The scale contains four response options and comprises two subscales of seven questions each. The subscales measure anxiety and depression, respectively, and range from 0 to 21, with a higher score denoting a greater number of symptoms of either anxiety or depression. For the purposes of this study, the instrument was translated into the native language of patients. The HADS scores were converted into a binary categorical variable. Depression was measured by using seven items of the depression subscale of HADS with cut-off points of ⩾8 and anxiety was measured by using seven items of the anxiety subscale HADS with cut-off points of ⩾8 [6, 9]. A review of the psychometric properties of HADS found that in most studies, an optimal balance between sensitivity and specificity was achieved when a case was defined by a score of 8 or above on both HADS-A and HADS-D [21].

### Data analysis

Data were analysed using SPSS version 24. Means, frequencies, proportions and rates of the given data for each variable were calculated. Single-variable analysis was done to characterise the association of each independent variable with the outcome variable. Those variables having *P*-value <0.2 were entered into the multivariable analysis by logistic regression model to identify the effect of each independent variable with the outcome variables. We conducted a backwards/forwards stepwise procedure when conducting the multivariable analysis. A *P*-value of <0.05 was considered statistically significant, and adjusted odds ratios with 95% CI were calculated to determine the association. Two sets of multivariable analysis by logistic regression analyses were run, using anxiety and depression as separate dependent variables to investigate correlates of anxiety and depressive symptoms independently.

### Ethical consideration

Ethical clearance was obtained from the University of Conakry. Participants were informed that the information collected for this research project would be kept confidential and information collected by this study would be coded with a unique identification number. HIV/AIDS patients who were found to have depression and anxiety were referred for further investigations and care if necessary.

## Results

### Socio-economic and demographic characteristics

A total of 160 participants were included in the study. Most of the study participants were female (118/160; 73.8%). The mean age of the study participants was 40.6 (±standard deviation 10.8) years. Participants were mostly living with a spouse (83/160; 51.9%), were private or governmental employees (122/160; 76.3%) and had some education (104/160; 65.0%). Demographic characteristics are shown in Table 1.

**Table 1.** Distribution of HIV/AIDS patients by socio-demographic factors at UTH in Conakry, 2018 (*n* = 160)

	Categories	Frequency	Per cent (%)
Sex	Male	42	26.3
	Female	118	73.8
Age	20–39 years	80	50.0
	⩾40 years	80	50.0
Education	Unable to read and write	56	35.0
	Primary and plus	104	65.0
Occupation	House wife/unemployment	38	23.8
	Private or governmental employee	122	76.3
Marital status	Divorced/widowed	53	33.1
	Single	24	15.0
	Married	83	51.9

### Clinical and psychosocial characteristics of the participants

Most of the study participants were recruited in the ambulatory clinic (146/160; 91.3%) and nearly all participants had commenced ART (155/160; 96.9%). The mean time duration from HIV diagnosis to study enrolment was 5.0 (±3.4) years. A little over half (94/160; 58.8%) of participants had CD4 count ⩽200 cells/μL at the time of diagnosis. Amongst all study participants, 12 (7.5%) had comorbid tuberculosis (Table 2), four had diarrhoea (2.5%) and one had Kaposi's sarcoma (0.6%). Over 13% (20/160) had temporarily stopped ART at least once in the last year.

**Table 2.** Distribution of clinical factors among HIV/AIDS patients at UTH in Conakry, 2018 (*n* = 160)

	Categories	Frequency	Per cent (%)
Patient status	Ambulatory	146	91.3
	Hospitalised	14	8.8
Body mass index	⩽18	32	20.0
	18–25	90	56.3
	>25	38	23.8
Duration of knowing HIV status	⩽5 years	91	56.9
	>5 years	69	43.1
Comorbid disease	No tuberculosis	148	92.5
	Tuberculosis	12	7.5
CD4 count at diagnosis	Missing	11	6.9
	<200 cells/mm^3^	55	34.4
	⩾200 cells/mm^3^	94	58.8
Started ART	Yes	155	96.9
	No	5	3.1
Temporary stop of ART	Yes	20	12.5
	No	135	84.4
	Never started	5	3.1

### Prevalence of depression and anxiety among HIV/AIDS patients

The prevalence of symptoms of anxiety was 13.8% (22/160) (95% CI 8.8–19.4) and for depression was 16.9% (27/160) (95% CI 10.6–23.1). The prevalence of depression and/or anxiety among HIV patients in this study was 22.5% (36/160) (95% CI 16.3–29.4). The prevalence of comorbid depression and anxiety among HIV patients in this study was 8.1%. Out of 27 patients whose HAD-D score indicated likely depression, 13 (13/160; 48.1%) reported feeling anxiety (Table 3). Meanwhile, nine (9/160; 6.8%) reported feeling anxiety of 133 whose HAD-D score suggested that they were not depressed.

**Table 3.** Agreement of depression and anxiety diagnosis based on HAD, among HIV patients at UTH in Conakry, 2018 (*n* = 160)

		Anxiety diagnosis based on HAD-A ⩾ 8		
		Absence, *n* (%)	Presence, *n* (%)	Total, *n* (%)
Depression diagnosis based on HAD-D ⩾ 8	Absence, *n* (%)	124 (93.2)	9 (6.8)	133 (86.3)
	Presence, *n* (%)	14 (51.9)	13 (48.1)	27 (16.9)
	Total, *n* (%)	138 (86.3)	22 (13.8)	160 (100)

*P*-value for *χ*^2^ test <0.001.

### Factors associated with depression and anxiety among patients with HIV/AIDS

Multivariate logistic regression analysis revealed that factors associated with depressive symptoms (HAD-D ⩾ 8) included individuals having BMI ⩽ 18 (AOR = 3.62, 95% CI 1.37–9.57) and participants who did not receive ART (AOR = 18.93, 95% CI 1.88–188.81). Similarly, patients aged <40 years had the odds 2.81 times greater to have anxiety as compared to patients aged ⩾40 years (AOR = 2.81, 95% CI 1.04–7.58) (Table 4).

**Table 4.** Factors associated with depression and anxiety among HIV patients at UTH in Conakry, 2018 (*n* = 160)

Factors		Depression (HAD-D ⩾ 8)				Anxiety (HAD-A ⩾ 8)			
		*n* (%)	Crude OR (95% CI)	*P* value	Adjusted OR (95% CI)	*n* (%)	Crude OR (95% CI)	*P* value	Adjusted OR (95% CI)
Sex	Male	6 (14.3)	1	0.60		3 (7.1)	1	0.16	
	Female	21 (17.8)	1.29 (0.48–3.47)			19 (16.1)	2.49 (0.69–8.90)		
Age	20–39 years	16 (20.0)	1.56 (0.67–3.63)	0.29		15 (18.8)	2.40 (0.92–6.26)	0.07	2.81 (1.04–7.58)
	⩾40 years	11 (13.8)	1			7 (8.8)	1		1
Education	Unable to read and write	9 (16.1)	1	0.84		6 (10.7)	1	0.42	
	Primary and plus	18 (17.3)	1.09 (0.45–2.62)			16 (15.4)	1.51 (0.55–4.12)		
Occupation	Unemployment	7 (18.4)	1.15 (0.44–2.97)	0.77		8 (21.1)	2.05 (0.78–5.36)	0.14	2.52 (0.92–6.87)
	Employee	20 (16.4)	1			14 (11.5)	1		1
Marital status	Divorced/widowed	8 (15.1)	1.16 (0.43–3.11)	0.08		10 (18.9)	1.91 (0.72–5.07)	0.42	
	Single	8 (33.3)	3.27 (1.13–9.44)			3 (12.5)	1.17 (0.29–4.73)		
	Married	11 (13.3)	1			9 (10.8)	1		
Body mass index	⩽18	12 (37.5)	3.55 (1.40–8.97)	0.003	3.62 (1.37–9.57)	7 (21.9)	2.24 (0.77–6.49)	0.33	
	18–25	13 (14.4)	1		1	10 (11.1)	1		
	>25	2 (5.3)	0.32 (0.07–1.53)		0.39 (0.08–1.85)	5 (13.2)	1.21 (0.38–3.81)		
Patient status	Ambulatory	22 (15.1)	1	0.06		18 (12.3)	1	0.10	
	Hospitalised	5 (35.7)	3.13 (0.95–10.22)			4 (28.6)	2.84 (0.80–10.02)		
Duration to know HIV status	⩽5 years	14 (15.4)	1	0.56		9 (9.9)	1	0.11	
	>5 years	13 (18.8)	1.27 (0.55–2.92)			13 (18.8)	2.11 (0.84–5.28)		
Started ART	No	4 (80.0)	22.95 (2.45–214.69)	0.006	18.93 (1.88–189.81)	1 (20.0)	1.59 (0.17–14.97)	0.68	
	Yes	23 (18.4)	1		1	21 (13.5)	1		
Temporary stop of ART	Never started	4 (80.0)	26.0 (2.75–245.89)	0.01		1 (20.0)	1.86 (0.19–17.69)	0.28	
	Yes	5 (25.0)	2.17 (0.70–6.69)			5 (25.0)	2.48 (0.79–7.74)		
	No	18 (13.3)	1			16 (11.9)	1		
Tuberculosis	No	22 (14.9)	1	0.03		19 (12.8)	1	0.25	
	Yes	5 (41.7)	4.09 (1.19–14.04)			3 (25.0)	2.26 (0.56–9.11)		

## Discussion

This study represents one of a few studies that systematically and simultaneously investigated correlates of symptoms of anxiety and depression in patients of public sector in a resource-limited setting. This study used the HADS scale, described as the best currently available to faithfully and validly assess anxiety and depression in HIV-infected patients [22].

Data on mental health in the general population are extremely limited in Africa, and thus comparable normative data for our tools were lacking. This study revealed that the prevalence of comorbid depression and anxiety in HIV-infected patients in Conakry, Guinea was 8.1%. Separately, 16.9% had depression and 13.8% had anxiety. Regarding the prevalence of depression, the current study result is line with other studies carried out in Tanzania [23], Ethiopia [24] and Ivory Coast [19], in which the prevalence estimates were reported to be 15.5%, 14.6% and 9.7%, respectively. On the other hand, the present study findings were lower than from studies done in South Africa, China and Nigeria in which the prevalence was reported to be 25.4%, 32.9% and 39.6%, respectively [9, 25, 26].

The prevalence of anxiety in this study was lower than the studies done in China, South Africa and Nigeria in which the prevalence was reported to be 27.4%, 30.6% and 32.6%, respectively [9, 25, 26]. We found a prevalence rate of 8.1% for comorbid anxiety and depression in this study among HIV-positive patients. The prevalence rate of comorbid anxiety and depression among the HIV-positive patients was far lower than a prevalence rate of 21.9% reported in another study in this environment [26]. However, similar prevalence has been reported in another study in which the prevalence was reported to be 5.3% [27]. The variation of the prevalence might be due to different diagnostic tools, sample size variations and different locations of the study.

The introduction of a standardised assessment of mental health into HIV services as HADS could be done and that could be a successful way to identify patients who could benefit from clinical evaluation given that these mental health issues are treatable. The guidelines of the WHO recommend that HIV-positive patient, their relatives and their caregivers receive psychosocial support [28]. Such psychosocial support may contribute to improvements in the health and treatment outcomes of HIV-positive people [29]. In Nepal, Pokhrel *et al*. showed that an intervention had positive effects in reducing depressive symptoms, anxiety and non-adherence to ART among people living with HIV at 6-month follow-up [30]. The intervention was comprised of home-based psychosocial support and peer counselling, adherence support, basic health care and referral services.

The present study found that having BMI ⩽ 18 was associated with depression. These findings are consistent with the findings from a study done at the Bamako University Hospital in Mali [31]. This may be because lower BMI in patients could be an indicator proxy marker of other comorbidities such as oral and pharyngeal candidiasis or a sicker patient overall.

HIV-untreated patients had 18.9 times higher odds of having depression as compared with those who are currently accessing ART. This association has also been reported by other researchers. Depression has been reported to be associated with delayed initiation of ART among the HIV-positive patients [32, 33]. ART can play an important role in preventing HIV transmission by reducing viral load to undetectable levels in the blood and other bodily fluids in infected persons. Early and sustained HIV treatment would provide this public health benefit and also reduce opportunistic infections and HIV/AIDS-related death, benefiting ART recipients [34, 35].

In this study, being younger resulted in the odds 2.81 times higher of having anxiety as compared to older (⩾40 years) patients. Younger age as a risk factor for psychiatric morbidity was also reported by other researchers [26, 27, 36].

## Limitations

Our study had several potential limitations. The cross-sectional study design did not allow for the assessment of the temporal relationship between anxiety/depression and factors. Our study recruited HIV patients from a single department, and hence, we cannot generalise to the entire HIV patient population in Guinea.

## Conclusions

In summary, there was a high prevalence of anxiety and depression symptoms among treatment-experienced HIV-infected individuals. Routine screening for depressive and anxiety symptoms is recommended with a proper integration of mental health services into HIV care to give comprehensive management to all HIV patients. The Guinea Ministry of Health should develop guidelines to screen and treat depression and anxiety among HIV patients. Further research on risk factors of depression and anxiety should be conducted to strengthen and broaden these findings in resource-limited settings.
